# Percentage of Tumor Invasion at Pretreatment High-Resolution Magnetic Resonance Imaging: Associating With Aggressive and Tumor Response in Chinese T3 Rectal Cancer-Preliminary Results

**DOI:** 10.3389/fonc.2022.616310

**Published:** 2022-04-07

**Authors:** Xiaoxin Hu, Jianwen Li, Yinan Sun, Yiqun Sun, Tong Tong

**Affiliations:** ^1^ Department of Radiology, Fudan University Shanghai Cancer Center; Department of Oncology, Shanghai Medical College, Fudan University, Shanghai, China; ^2^ Department of Urology, Dushu Lake Hospital Affiliated To Soochow University, Medical Center of Soochow University, Suzhou Dushu Lake Hospital, Suzhou, China; ^3^ Department of Propaganda，Shanghai Yangpu District Central Hospital, Shanghai, China

**Keywords:** rectal neoplasms, magnetic resonance imaging, feasibility studies, neoplasm staging, chemoradiotherapy

## Abstract

**Purpose:**

The purpose of the study was to assess the ability of percentage of tumor invasion (PTI) of T3 rectal cancer on pretreatment MRI as an imaging biomarker to reflect aggressiveness and to predict tumor response after neoadjuvant chemoradiation (NCRT) in Chinese population.

**Methods:**

A total of 107 Chinese rectal cancer patients who underwent pretreatment MRI staging as T3 were included. The extramural depth of tumor invasion (EMD), the distance between outer border of muscularis propria (MP) and mesorectal fascia (MRF) we called “thickness of the mesorectum (TM)”) at the same slice and direction were measured at pretreatment MRI, and PTI was equal to EMD/TM, was calculated. The EMD and PTI of subgroups based on pretreatment CEA, CA19-9 levels; N category and pathological complete response (pCR) were compared. The parameters, which described tumor invasion, were compared between pCR and non-pCR group. Student t-tests and logistic analysis were applied.

**Results:**

The pretreatment PTI was higher in CEA ≥5.2 ng/ml patients (58.52% ± 27.68%) than in CEA <5.2 ng/ml patients (47.27% ± 24.15%) (*p* = 0.034). The pretreatment EMD in non-pCR group (7.21 ± 2.85 mm) was higher than in pCR group (6.14 ± 3.56 mm) (*p* = 0.049). The pretreatment PTI in non-pCR group (57.4% ± 26.4%) was higher than in pCR group (47.3% ± 29.1%) (*p* = 0.041). Compared with patients with PTI ≥50%, MRF (+), more patients with PTI <50%, MRF (−) showed pCR (OR = 8.44, *p* = 0.005; OR = 6.32, *p* = 0.024).

**Conclusion:**

The PTI obtained at pretreatment MRI may serve as an imaging biomarker to reflect tumor aggressiveness and predict which T3 rectal cancer patients may benefit from NCRT in Chinese population.

## Introduction

Neoadjuvant chemoradiation (NCRT) followed by surgery for rectal cancer patients with T3 stage were recommended by the NCCN Clinical Practice Guidelines (1, 2). The 5-year survival rate of patients with T3 stage varying from 30 to 80% have demonstrated with obviously heterogeneous prognosis, indicating the need for these patients to further stratification before treatment by noninvasively imaging methods (3–5).

Extramural depth of tumor invasion (EMD) has been shown to be an independent risk factor for recurrence in rectal cancer (6, 7). The cancer-specific survival rate drops from 85 to 54%, independent of nodal involvement, when EMD exceeds 5 mm (8). The definitions of subcategory T3a–T3d based on EMD (T3a: <1 mm, T3b:>1–5 mm, T3c: >5–15 mm, T3d: >15 mm) according to the European Society for Medical Oncology (ESMO) Clinical Practice Guidelines (9). However, in clinical practice, intra- and inter-observer reproducibility for the measurements of EMD is poor and the EMD less than 1 mm is especially difficult to measure at pretreatment magnetic resonance imaging (MRI) (10, 11). It is not uncommon to encounter early T3 (T3a/b) disease with threatened MRF at pretreatment MRI in Chinese population, because of the thin mesorectum surrounding the lower rectum. Therefore, the use of the EMD and T3 subcategory should be discussed in Chinese population. Our study aimed to evaluate whether the percentage of tumor invasion (PTI), which was equal to EMD/thickness of mesorectum (TM) at pretreatment MRI, could reflect the aggressiveness of tumor and predict the tumor response to NCRT in T3 rectal cancer.

## Materials and Methods

### Patients

**Figure 1:**
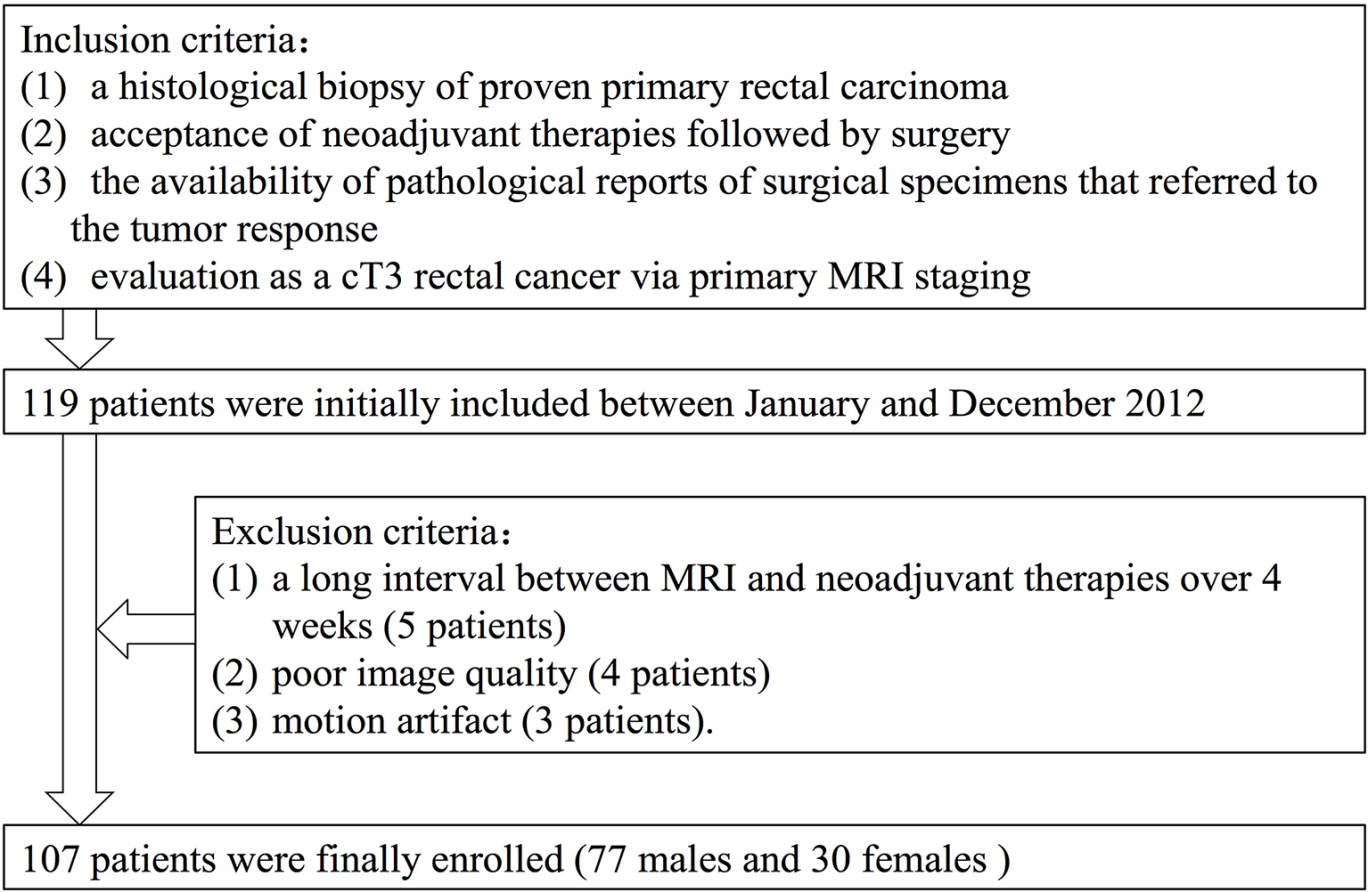
Flowchart of the study population.

Between January and December 2012, 107 rectal cancer patients who were diagnosed and treated at the Fudan Cancer Hospital were selected as subjects in this retrospective study (**Figure 1**). Selection criteria included the following: 1) a histological biopsy of proven primary rectal carcinoma; 2) acceptance of NCRT followed by surgery; 3) the availability of pathological reports of surgical specimens that referred to the tumor response, and 4) evaluation as a T3 rectal cancer at pretreatment MRI staging. Our initial analysis identified 119 patients who matched the above criteria. Exclusion criteria included 1) a long interval between MRI and NCRT over 4 weeks (five patients), 2) poor image quality (four patients), and 3) motion artifact (three patients).

Clinical and imaging data were retrieved from the patient database. This study was approved by the Medical Ethics Committee of the Fudan University Shanghai Cancer Center (Shanghai, China), and was performed according to the principles of the Declaration of Helsinki. All subjects or guardians provided signed informed consent.

The final study population consisted of the 107 remaining patients (seventy-seven males and thirty females). The average study age was 57 years, with a range of 28–84 years. Sixty-four patients received low anterior resection (LAR), thirty-six patients underwent abdomino-perineal-resection (APR) and seven patients received Hartmann surgery.

### MR Imaging

Pretreatment MRI was performed on a 3.0 Tesla (T) MR magnet (Signa Horizon, GE Medical Systems, Milwaukee, WI) using a phased-array body coil. Sagittal T2-weighted (T2W) fast spin echo [repetition time/echo time (TR/TE): 2,540/100 ms, echo train length: 16, field of view (FOV):16 cm, section thickness: 3 mm, interspace: 0.5 mm, number of slices: 16, number of excitations (NEX):1, matrix: 224 × 320] and oblique axial thin-section T2W [repetition time/echo time (TR/TE): 3,420/110 ms, flip angle: 90°, echo train length: 20, field of view (FOV): 20 cm, section thickness: 3 mm, interspace: 1 mm, number of slices: 20, number of excitations (NEX): 2, matrix: 384 × 224] were used for this investigation. Axial diffusion-weighted images (DWI) were obtained using the following parameters: b-values: 0, 800 s/mm², TR/TE: 2,800/67 ms, echo planar imaging [EPI] factor: 53, number of slices: 28 slices, and acquisition time: 2 min 30 s. Enhanced images were acquired after the intravenous administration of gadopentetate dimeglumine (Gd-DTPA, Magnevist, Bayer Health-Care Pharmaceuticals Products, Germany) using axial LAVA sequence (TR/TE: 3.4/1.5 ms, flip angle: 15°, FOV: 33 cm, section thickness: 4.8 mm, interspace: 0 mm, number of slices: 38, acquisition time: 19 s). Patients did not receive bowel preparation antispasmodic medication or rectal distention before the MR examinations.

### Extramural Depth of Tumor Invasion, Thicknesses of Mesorectum, Percentage of Tumor Invasion

**Figure 2:**
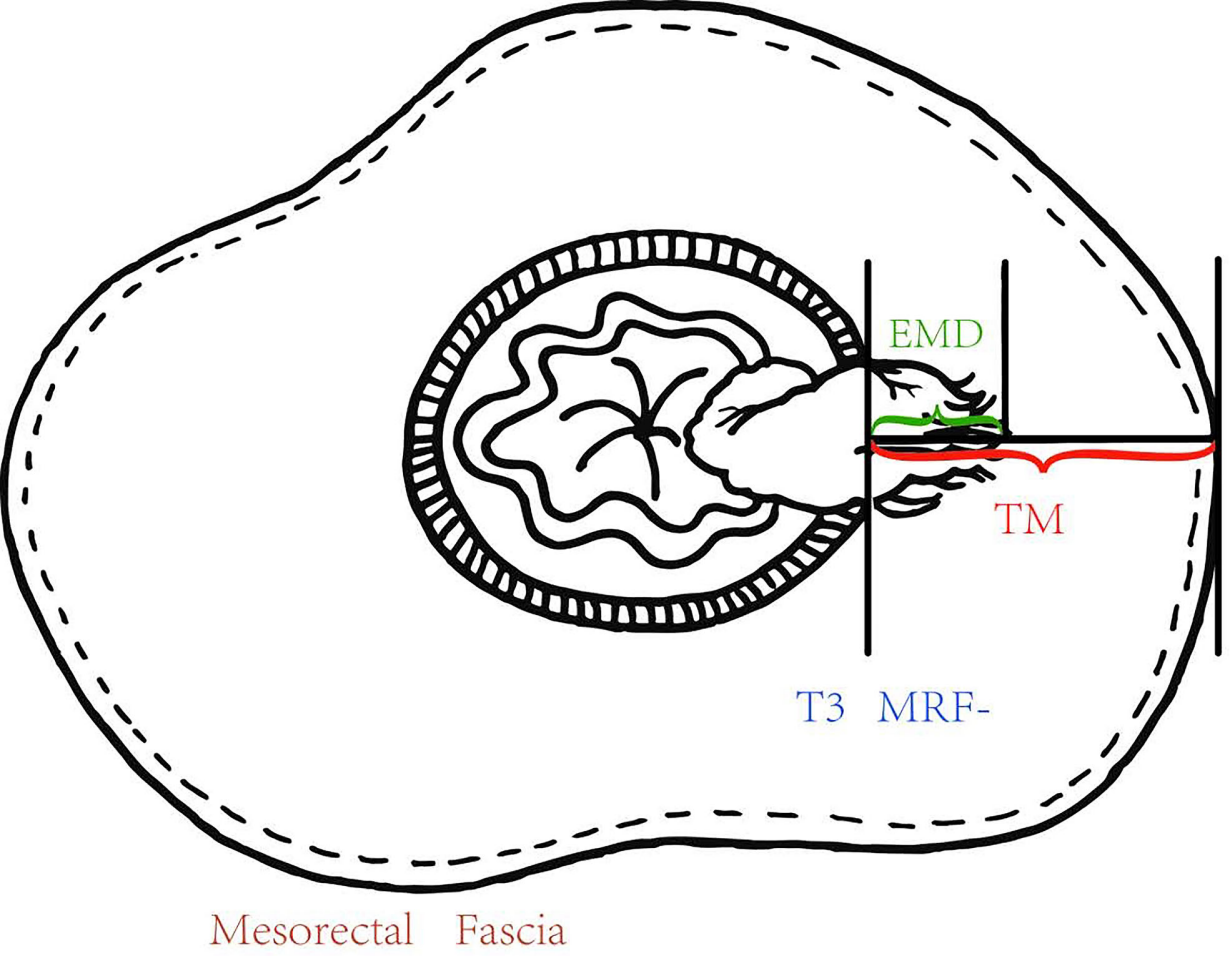
The EMD was measured and recorded using the calipers of workstation, and the TM from the outer edge of the MP to the MRF according to the same path of EMD and the PTI which was equal to EMD/TM were calculated.

The pretreatment MRI images were independently reviewed on a picture archiving and communication system (PACS) by two gastrointestinal radiologists. The two gastrointestinal radiologists were blinded to the information obtained at surgery and the pathological analysis. To avoid any recall bias, the order of cases was changed in each review session. One professor had more than 15 years of experience, and the less-experienced professor had 5 years of experience in interpreting abdominal MRI images. Each radiologist used a workstation to interpret the images and to identify the image that depicted the maximal extramural tumor spread. For each patient, the EMD was measured and recorded using the calipers of workstation. The TM, which was from MP to MRF according to the same path of EMD was measured (**Figure 2**). EMD and TM were taken from the average measurement of the two radiologists. PTI value was EMD divided by TM.

### Prognostic Factors

The clinical factors examined were the plasma CEA (ng/ml) and CA19-9 (U/ml) levels at the same day, of which pretreatment MRI was performed. Patients with CEA <5.2 ng/ml was considered normal CEA levels. Patients with CEA ≥5.2 ng/ml was considered elevated CEA levels (threshold used in our institution). Patients with CA19-9 <35.5 U/ml was considered normal CA19-9 levels. Patients with CA19-9 ≥35.5 U/ml was considered elevated CA19-9 levels (threshold used in our institution). Nodes larger than 9 mm and nodes are always regarded as cN (+). Smaller lymph nodes require additional morphologically suspicious features (round shape, indistinct border, heterogeneous signal) in order to be considered as cN (+). The MRF is considered involved when the distance between the tumor margin and MRF is less than 1 mm. The distance between the anal verge and the tumor was calculated as a sum of series of small straight-line segments at pretreatment MRI. When there was disagreement between the two radiologists, a third radiologist would reanalyze the imaging data, and the majority opinion was accepted.

### Treatment

All patients received NCRT. Radiotherapy (RT) was delivered with a linear accelerator using 6- and 15-MV photons and a three-field technique (posterior–anterior and right and left laterals). Every patient underwent a planning CT scan in the treatment position (prone position) using a belly board. Three-dimensional conformal RT was used for all patients based on the planning CT, with a total dose of 45 Gy at 1.8 Gy per fraction per day, Monday–Friday. NCRT was delivered concurrently with RT. Starting on day 1 of RT, patients received capecitabine 625 mg/m^2^ orally, twice a day (Monday–Friday), and oxaliplatin 50 mg/m^2^ weekly for five consecutive weeks. Surgery was scheduled eight weeks after the completion of NCRT. Total mesorectal excision (TME) was mandatory, whereas the form of surgery (anterior resection or abdominal-perineal resection) and whether a temporary colostomy should be performed were decided by the surgeon.

### Histopathologic Evaluation

The most commonly used method to assess the rectal tumor response after NCRT is the pathological complete response (pCR) and non-pCR. pCR is defined as the absence of viable adenocarcinoma cells in the surgical specimen. In the present study, we classified the patients into pCR and non-pCR groups.

### Statistical Analysis

Interclass agreements of EMD and TM were calculated from the measurements of the two radiologists. An interclass correlation coefficient greater than 0.75 indicated a good agreement.

The PTI value was EMD divided by TM (both EMD and TM were taken as the average measurement of the two radiologists). Student t-tests (independent-samples t-test) were used to assess the differences between means of the following groups: cN (−) and cN (+); CEA <5.2 ng/ml and CEA ≥5.2 ng/ml; CA19-9 <27 U/ml and CA19-9 ≥27 U/ml (threshold used in our institution); pCR and non-pCR. A logistic analysis was applied to identify independent variables influencing the tumor response.

Statistical analyses were performed using the Statistical Package for the Social Sciences (SPSS 16.0, SPSS, Chicago, Ill). For all the above-mentioned analyses, a *p*-value of less than 0.05 was considered statistically significant.

## Results

### Patient Characteristics

The tumor characteristics of the 107 rectal cancer patients are listed in **Table 1**.

**Table 1:** Patient Characteristics.

		Total
**Gender**	Male	77
	Female	30
**Age**	All	58 (28–84)
	T3a	0
**T3 subcategory**	T3b	30
	T3c	76
	T3d	1
**cN category**	(-)	9
	(+)	98
**MRF status**	(-)	77
	(+)	30
Direction^*^	Anterior	9
	Lateral	90
	Posterior	8
Length (cm)^**^	<5	61
	≥5	46
Distance (cm)^***^	<5	71
	≥5	36
**Operation**	APR	36
	LAR	64
	Hartmann	7
**CEA** **(ng/ml)**	<5.2	77
	≥5.2	30
**CA19-9** **(U/ml)**	<27	85
	≥27	22
**EMD (mm)**	All	7.01 ± 3.01
**TM (mm)**	All	14.95 ± 7.43
**PTI (%)**	All	55.37 ± 27.11
**Tumor response**	pCR	20
	Non-pCR	87

Direction^*^, The main direction of tumor invasion.

Length^**^, The tumor length measured at MRI.

Distance^***^, The distance from the anal verge to the edge of the tumor.

EMD, The extramural depth of tumor invasion; PTI, The percentage of tumor invasion; MRF, mesorectal fascia; CEA, carcinoembryonic antigen; CA19-9, carbohydrate antigen 19-9; APR, abdomino-perineal-resection; LAR, low anterior resection.

### Quantitative Analysis

#### Difference of EMD, T3 Subcategory and PTI Based on the Prognostic Factor in T3 Rectal Cancer

The interclass correlation coefficients of the measurements of the two radiologists about EMD and TM were 0.853 and 0.877. The mean EMD was 7.01 ± 3.01 mm, the mean TM was 14.95 ± 7.43 mm, and the mean PTI was 55.37 ± 27.11%. **Table 2** presents the differences in EMD and PTI values between the different subgroups. The PTI was significantly higher in CEA ≥5.2 ng/ml patients (58.52% ± 27.68%) than in CEA <5.2 ng/ml patients (47.27% ± 24.15%) (*p* = 0.034). We compared EMD and PTI values between the pCR group and non-pCR group. Before NCRT, the mean tumor EMDs in non-pCR group (7.21 ± 2.85 mm) was significantly higher than in pCR group (6.14 ± 3.56 mm) (*p* = 0.049). The mean PTI in the non-pCR group was significantly higher than in the pCR group (*p* = 0.041).

**Table 2:** Difference of EMD, PTI and T3 subcategory according to the cN category, CEA, CA19-9 and pCR in T3 rectal cancer.

		EMD (mm)	*p*	PTI (%)	*p*	T3 subcategory		*p*
						a + b	c + d	
**cN**	(−)	5.93 ± 4.59	0.26	55.27 ± 26.48	0.14	1	8	0.260
**category**	(+)	7.11 ± 2.84		56.44 ± 35.12		29	69	
**CEA** **(ng/ml)**	<5.2	6.76 ± 3.07	0.17	47.27 ± 24.15	0.034	26	51	0.468
	≥5.2	7.65 ± 2.80		58.52 ± 27.68		4	26	
**CA19-9 (U/ml)**	<27	6.82 ± 2.97	0.20	54.5 ± 23.12	0.87	28	57	0.209
	≥27	7.75 ± 3.11		55.59 ± 28.17		2	20	
**Tumor**	pCR	6.14 ± 3.56	0.049	47.3 ± 29.1	0.041	4	16	0.118
**Response**	Non-pCR	7.21 ± 2.85		57.4 ± 26.4		26	61	

EMD, The extramural depth of tumor invasion; PTI, The percentage of tumor invasion; pCR, Pathological complete response.

#### Factors Influencing the Tumor Response of T3 Rectal Cancer

**Figure 3:**
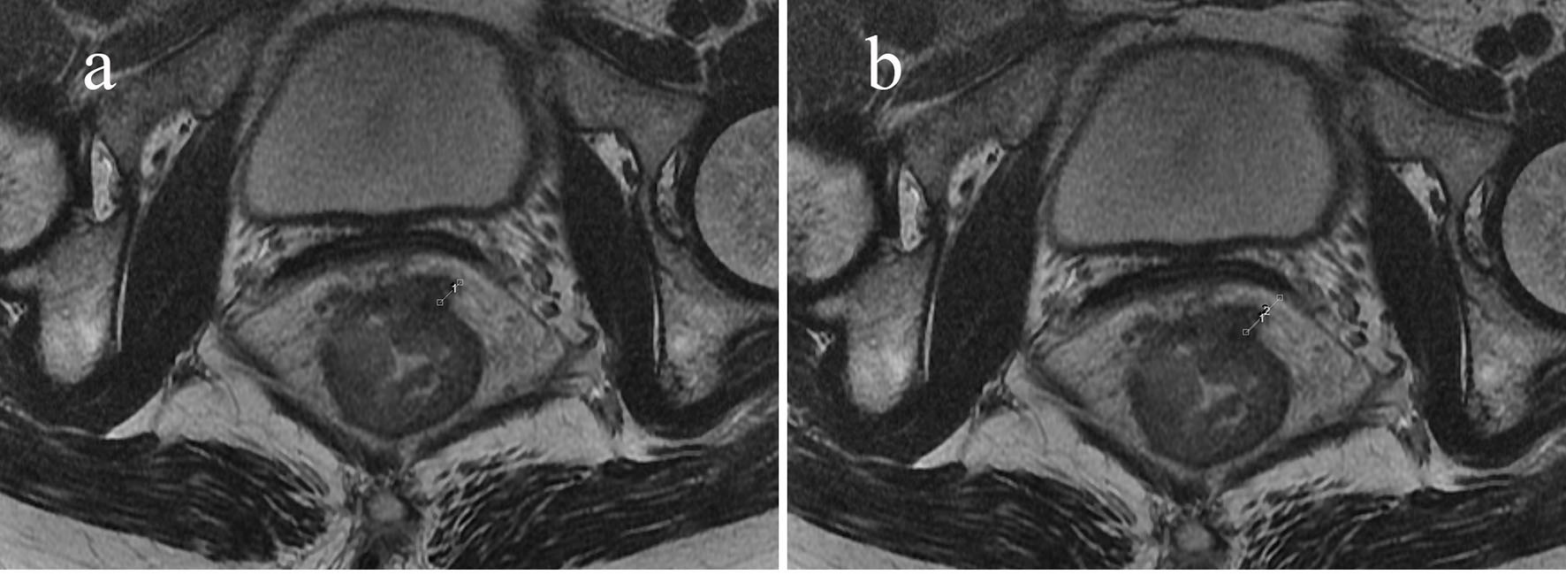
Sixty-three-year-old woman with an mr-T3N0 rectal adenocarcinoma 5 cm from the anal verge. Pretreatment rectal MRI **(A, B)** before NCRT. The EMD was 6.1 mm **(A)**, TM was 10.5 mm **(B)**, and the calculated PTI was 58.1%. The rectal cancer had slightly regressed after NCRT. The postoperative staging confirmed by pathology was T3N0, and the tumor response was non-pCR.

**Figure 4:**
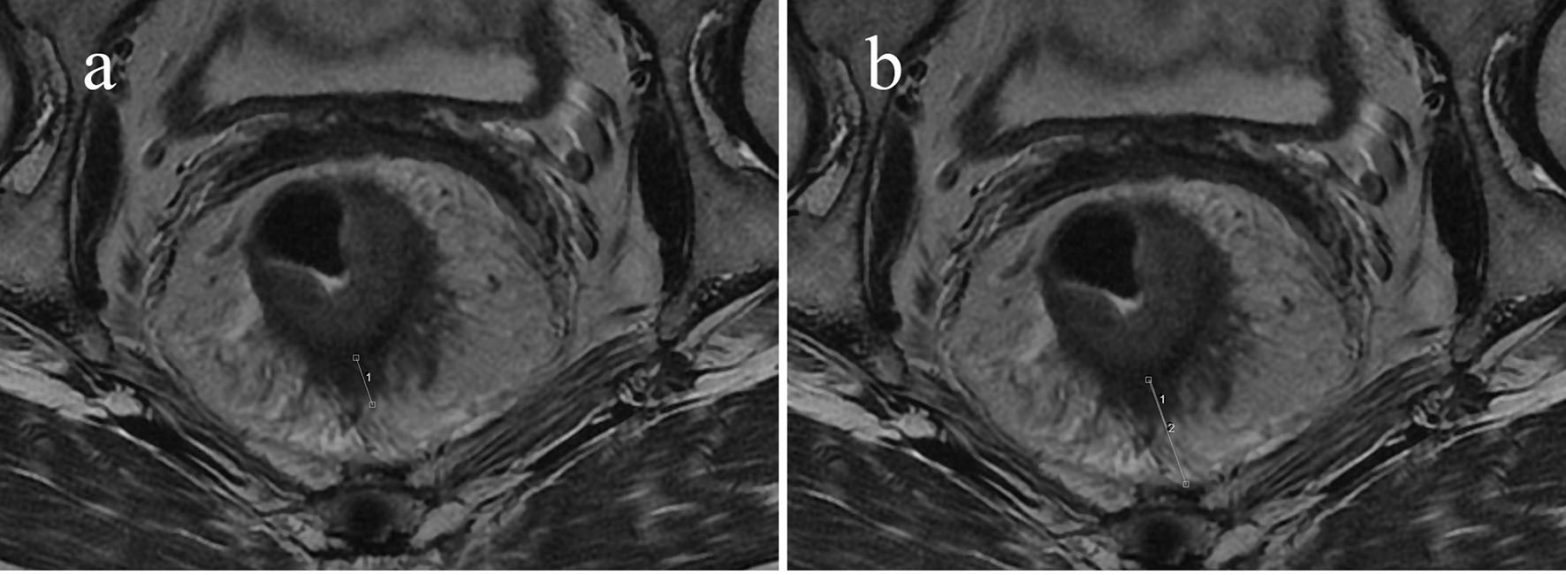
Sixty-seven-year-old man with an mr-T3N0 rectal adenocarcinoma 9.5 cm from the anal verge. Pretreatment rectal MRI **(A, B)** before NCRT. The EMD was 9.7 mm **(A)**, the TM was 21.7 mm **(B)**, and the calculated PTI was 44.7%. The rectal cancer had regressed after NCRT. The postoperative staging confirmed by pathology was T0N0, and the tumor response was pCR.

Factors influencing the tumor response of T3 rectal cancer were PTI (*p* = 0.001) and MRF status (*p* = 0.026), as determined by univariate analysis. Other factors, such as gender, EMD, cT3 subcategory, cN category, the main direction of tumor invasion, the length of tumor, CEA and CA19-9 level did not influence the tumor response. Further multivariate analysis showed that factors influencing the tumor response were PTI and MRF. Compared with patients with PTI ≥50% and MRF (+), significantly more patients with PTI <50% and MRF (−) showed pCR (OR = 8.44, *p* = 0.005; OR = 6.32, *p* = 0.024, respectively) (**Figures 3** and **4**). The above results are shown in **Tables 3** and **4**.

**Table 3:** Univariate Analysis in Factors Influencing pCR of T3 Rectal Cancer.

		Patient		X^2^	*P*
		pCR	Non-pCR		
**Gender**	Male	15	62		
	Female	5	25	0.112	0.737
**EMD (mm)**	EMD	6.14 ± 3.56	7.21 ± 2.85	76.513	0.150
**T3**	T3a + b	4	26		
**subcategory**	T3c + d	16	61	4.274	0.118
**PTI (%)**	<50	15	42		
	≥50	5	45	14.665	0.001
**cN category**	(−)	4	5		
	(+)	16	82	1.745	0.187
**MRF status**	(−)	17	60		
	(+)	3	27	8.318	0.026
Direction^*^	Anterior	1	8		
	Lateral	18	71		
	Posterior	1	7	0.643	0.725
Length^**^ **(cm)**	<5	13	48		
	≥5	7	39	0.641	0.423
Distance^***^ **(cm)**	<5	15	56		
	≥5	5	31	5.842	0.054
**CEA** **(ng/ml)**	<5.2	5	25		
	≥5.2	15	52	0.303	0.582
**CA19-9** **(U/ml)**	<27	3	19		
	≥27	17	68	1.913	0.167

Direction^*^, The main direction of tumor invasion; Length^**^, The tumor length measured on MRI; Distance^***^, The distance from the anal verge to the edge of the tumor; EMD, The extramural depth of tumor invasion; PTI, The percentage of tumor invasion; MRF, mesorectal fascia.

**Table 4:** Multivariate Analysis in Factors Influencing pCR of T3 Rectal Cancer.

		Patient		OR	95%CI	P
		PCR	Non-pCR			
**PTI (%)**	<50	15	42			
	≥50	5	45	8.44	(1.90–37.50)	0.005
**MRF status**	(−)	17	60			
	(+)	3	27	6.32	(1.49–26.7)	0.024

EMD, The extramural depth of tumor invasion; PTI, The percentage of tumor invasion; OR, Odds ratio; CI, Confidence interval.

## Discussion

In clinical practice, we found that the TM was varied in different directions and slices. Furthermore, it was not uncommon to see a T3a/b tumor with threatened MRF as evaluated at pretreatment MRI in the Chinese population. A recent report also proposed that the use and limitation of the T3 subcategory in the Chinese population should be discussed (12). Two studies have shown the EMD/mesorectum ratio may serve to predict decreased recurrence free survival and disease-free survival in T3 rectal cancer patients respectively (11, 13). Our study was to assess the ability of PTI at pretreatment MRI as a potential noninvasive imaging biomarker to simultaneously reflect aggressiveness and predict the tumor response after NCRT. The N category, CEA, CA19-9 level, and tumor response are usually an indication of biological tumor aggressiveness (14–17). Therefore, we compared the EMD and PTI values between the cN (−) and cN (+) group, CEA (<5.2 ng/ml) and CEA (≥5.2 ng/ml) group, CA19-9 (<27 U/ml) and CA19-9 (≥27 U/ml) group, and pCR vs non-pCR group, respectively. EMD exhibited significant differences among subgroups with pCR and non-pCR; PTI also exhibited significant differences among subgroups with CEA <5.2 ng/ml and CEA ≥5.2 ng/ml, with pCR and non-pCR, respectively. The results showed that greater PTI was associated with CEA ≥5.2 ng/ml subgroup. This finding could be explained by the fact that the PTI values may reflect the aggressiveness of the tumor tissue profile, which was further supported by the finding that non-pCR would show a relatively high PTI. We also found that the EMD of non-pCR patients was deeper than patients who got pCR, suggesting that EMD by itself reflects the prognosis of T3 rectal tumor. Tong et al. (18) analyzed 90 pretreatment rectal MRI to determine whether the MRI assessment of EMD was associated with CEA, CA19-9 and the N category. This study did not include the PTI association between tumor responses, and the number of cases or population distribution was different. The EMD and PTI findings at pretreatment MRI were therefore able to reflect the biological behavior of rectal cancer and might be a potentially powerful imaging biomarker.

The findings raised interest in the possibility of a non-operative treatment strategy, the “watch-and-wait” approach, for rectal cancer (19). With the watch-and-wait approach, patients who achieve a clinical complete response (cCR) have organ-preserving nonsurgical treatment and are followed closely clinically and radiographically (20). The guideline regards MRI as one of the methods to evaluate cCR. However, challenges to this approach include identifying which patients would benefit from the “watch-and-wait” approach. More-accurate explorations are necessary to offer non-operative management to all patients who may benefit. Our study showed that the MRF status and PTI might be useful factors for predicting the pCR patients with rectal cancer receiving the NCRT. The MRF status at MRI corresponded to the circumferential resection margin (CRM), which was a strong risk factor for local recurrence and an established prognostic factor (21, 22). Our results also showed that MRF (+) served as a risk factor for pCR patients (OR = 6.32, *p* = 0.024), and most scholars accept that the NCRT effect and the long-term prognosis of patients are significantly correlated (23, 24). PTI ≥50%, as defined in this study and correctly identified, was another significant risk factor for pCR (OR = 8.44, *p* = 0.005). In addition to the well-known predictors, our study has shown that PTI might also be one of the predictors to tumor response in T3 rectal cancer. However, large-scale prospective studies and a validation study are warranted to confirm the predictive and prognostic significance of the PTI.

This study had several limitations: First, the number of patients included in the study was relatively small after being classified, and that this was a single-center study. Second, biases could be present in our cohort. There was no T3a patient, because T3a and T2 were difficult to distinguish and the distance less than 1 mm was harder to measure accurately. Also, only one patient staged T3d due to paucity of mesorectal fat in Chinese patients. Third, we chose to correlate the EMD and PTI with factors measured from the pretreatment MRI because we aimed to explore the correlation between the EMD, PTI and the primary tumor profile before it was affected by any therapeutic interventions. Hence, we could not use the pathological N-category for studying. The patients had already accepted chemoradiation therapy before surgery, in which case the N category at histology was no longer representative of the initial tumor profile. Fourth, it would have been clinically interesting to assess the aggressiveness profile of the tumors using outcome parameters such as disease-free or overall survival. However, this would require a larger patient cohort and a longer follow-up period, which was beyond the scope of our current study.

Despite these limitations, this study was informative. Pretreatment PTI values obtained directly from a routine rectal MRI can serve as a new noninvasive imaging biomarker that might be helpful in predicting the pCR of rectal cancers in Chinese population.

## Data Availability Statement

The original contributions presented in the study are included in the article/supplementary material. Further inquiries can be directed to the corresponding authors.

## Ethics Statement

We obtained essential approval from the Fudan University Shanghai Cancer Center Institutional Review Board (Shanghai, China); informed consent was obtained from all patients included in the study.

## Author Contributions

All authors listed have made a substantial, direct, and intellectual contribution to the work and approved it for publication.

## Funding

This study was supported by Shanghai Sailing Project (19YF1409900) and the National Natural Science Foundation of China (Grant No. 82001776,81971687). The funder played no role in the study design, data collection and analysis, decision to publish, or preparation of the manuscript.

## Conflict of Interest

The authors declare that the research was conducted in the absence of any commercial or financial relationships that could be construed as a potential conflict of interest.

## Publisher’s Note

All claims expressed in this article are solely those of the authors and do not necessarily represent those of their affiliated organizations, or those of the publisher, the editors and the reviewers. Any product that may be evaluated in this article, or claim that may be made by its manufacturer, is not guaranteed or endorsed by the publisher.
